# The Use of Social Network in Daily Pediatric Practice and Education: Turkish Pediatric Atelier

**DOI:** 10.1155/2020/7301309

**Published:** 2020-09-21

**Authors:** Erdem Gönüllü, Ahmet Soysal, İlkay Can, Ercan Tutak, Turan Tunç, İsmail Yıldız, Osman Yeşilbaş, Naci Öner, Ali Anarat, Feryal Gün Soysal, Cengiz Bayram, Mehmet Cihan Balcı, Emine Betül Tavil, Teoman Akçay, Metin Karaböcüoğlu

**Affiliations:** ^1^Üsküdar University Medical School, Dept. of Pediatrics Site yolu, Cd No: 27 34768 Ümraniye, İstanbul, Turkey; ^2^Memorial Ataşehir Hospital, Dept. of Pediatrics, Vedat Günyol Cd. No. 28, 34758 Ataşehir, İstanbul, Turkey; ^3^Burhaniye State Hospital, Dept. of Dermatology, Hürriyet Mah. Ali Kemal Deveci Bulvarı No: 106 10700 Burhaniye, Balıkesir, Turkey; ^4^Memorial Şişli Hospital Dept. of Pediatrics, Piyalepaşa Blv. 4, 34385 Şişli, İstanbul, Turkey; ^5^Karadeniz Technical University Medical School, Dept. of Pediatric Intensive Care, Trabzon, Turkey; ^6^İstanbul Technology and Health University, Dept. of Pediatric Cardiology, Seyitnizam, Mevlana Cd. No: 85, 34015 Zeytinburnu/İstanbul, Turkey; ^7^Memorial Ataşehir Hospital, Dept. of Pediatric Surgery, Vedat Günyol Cd. No. 28, 34758 Ataşehir İstanbul, Turkey; ^8^Hasan Kalyoncu University Faculty of Law, 34235 Şehitkamil Gaziantep, Turkey; ^9^İstanbul University, İstanbul Medical School, Dept. of Pediatric Metabolic diseases, Turgut Özal Millet Cd, 34093 Fatihİstanbul, Turkey

## Abstract

Using social media applications in pediatric education is not outdated, and its effectiveness has not been tested yet. For this reason, we shared the first results of the Pediatric Atelier experience that we realized through telegram application. We make an online survey to investigate the needs, requirements, pleasure, and suggestions of members through a web-based questionnaire. This cross-sectional survey study was delivered only to participants who were members of the workshop via their email addresses. Online questionnaires organized using Google Forms were sent to pediatric workshop members between March and June 2019. The questionnaire consisted of questions that measured the participants' basic demographic data, the use of the workshop, and the overall impact of the workshop on their professional behavior. While the institutions and positions of the participants were recorded, no other personal data (such as address and telephone) were collected. Among the 997 members, 417 (42%) of them answered the questionnaire. Respondents included 300 (72%) pediatrician, 21 (5%) pediatric subspeciality fellows, and 75 (18%) pediatric subspecialists. Of the 417 respondents, 217 (52%) were working in Istanbul, and 200 (48%) were working in other cities of Turkey. Among the responders, 233 (56%) were working in private hospitals or doctor offices. A total of 520 cases were consulted in 241 days of study period. Most consultations (*n* = 309, %59) were made from the Istanbul metropolitan area, and 203 (40%) consultations were from other cities of Turkey. The most frequently consulted departments were Pediatric infectious diseases: 166 (32%), Pediatric hematology and oncology: 56 (11%), and Neonatology: 43 (8%). Of the 520 consulted cases, 44 (8%) were related to life-threatening events, and 25 of them were hospitalized in the intensive care units, and 6 of them were required surgical operations. Of the 94% of responders thought this platform was useful and 82% of them stated that the case counseling part of the atelier was the most useful part. We think that the development of technology and artificial intelligence may lead to the usage of on-line platforms or systems in clinical medical practice. *Clinical Trial Registration (if any)*. Registry name, registration number, web link to study on registry, and data sharing statement.

## 1. Introduction

In the history of humankind, few technologies have resulted in such widespread social and economic change in a relatively short period of time as the Internet has. Growing nearly 900%, from 400 million users in 2000 to more than 4 billion users in 2019, the Internet has had an unprecedented impact on economies and societies around the globe [1]. Internet and smartphone applications are being used with increasing frequency by both patients and physicians in modern medical practice. It has been reported that e-learning produces acceptable results in postgraduate medical education [2]. A recent meta-analysis of 14 studies integrating social media (SM) platforms in medical education showed that interventions are associated with improved knowledge, attitudes, and increased student participation, feedback, collaboration, and professional development rates [3]. A survey of 207 medical oncologists reported that more than 50% of respondents were interested in using SM for professional purposes, but 59% said a barrier to doing so is not having enough time [4]. The survey responses of a group of European retina experts who exchanged information through SM were particularly more satisfied with SM than the group that could not find the opportunity to train due to being busy working was [5]. Training through SM applications in pediatric practice is a new topic. As far as we know, there are no studies related to the usage of social media (e.g., the Telegram application) in daily postgraduate pediatric practice. We organized the Turkish Pediatric Atelier Telegram group in October 2018. The aim of this study is to share our experience using Telegram groups in daily pediatric practice, postgraduate education, and pediatric consultations. Our secondary aim is to understand the need and benefits of such a heterogeneous group by using a questionnaire.

## 2. Materials and Method

The Turkish Pediatric Atelier group was first established by authors AS, EG, ET, TT, CÖ, and MK on the Telegram application on October 31, 2018. The group consists mainly of pediatricians, pediatric subspecialists, pediatric surgeons, child psychiatry specialists, and physicians serving for the pediatric population, including those specializing in pediatric dermatology, pediatric ear nose throat (ENT), pediatric radiology, and pediatric urology as well as an advocate—a pediatrics specialist for issues about pediatric malpractice and other medicolegal cases. Group members are included by invitation, and membership is confirmed by Telegram group directors. The Turkish Pediatric Atelier group is set up so that all members can ask questions related to pediatrics, radiological images, dermatologic conditions in patients, laboratory results, drug dosages, etc. To answer the questions from the members, we created a weekly rotation list of academic physicians to convey the queries to the relevant subspecialist and ensure that it is answered if the question is related to a specific subspecialty or if the question has not yet been answered. The members of our group assisted the physicians who requested patient transfer to other units for intensive care, advanced workup, and surgical intervention for their patients. For all data and images shared through Telegram, we require that the patient name be hidden and his or her face be blurred in accordance with laws on the protection of personal information. Each member must ask patients or caregivers to give verbal consent for their information or their child's information, respectively, to be shared. In addition, for educational purposes, on Mondays, for the “diagnosis of the day,” we share a short case history and ask members what their diagnoses are. On Tuesday, for the “image of the day,” we share an image of cases and ask what their diagnoses are. On Wednesday, for the “article of week,” we summarize newly released articles related to pediatrics, and on Thursday, for the “case of week,” we present a case through Telegram and interactively discuss which tests should be used for follow-up, which should be kept in mind in the differential diagnosis, what should be done next, and what the diagnosis and treatment are. Then, the exact diagnosis of cases and brief information about cases are given. Moreover, every month, we present a special topic through YouTube channel as live, interactive broadcasts, and simultaneously moderate a question and answer activity from the Telegram group.

Eight months after the Pediatric Workshop group was founded, we designed a descriptive survey study to investigate members' needs, requirements, wishes, and suggestions using a web-based survey. The survey preliminary form was shared in a group of 20 pediatricians who did not design this questionnaire before the study; all of the small group agreed that all items of the questionnaire were clearly understandable. As we did a descriptive study of the whole group, we did not perform reliability and validity assessment other than this validation.

**Figure 1:**
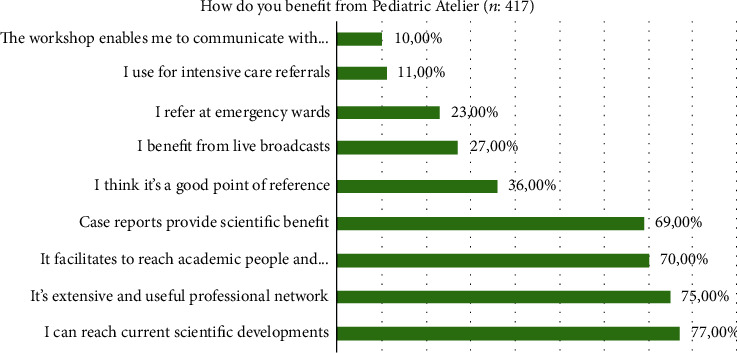
Member's opinions about Pediatric Atelier.

This cross-sectional survey study was delivered only to participants who were members of the workshop via their email addresses. Online questionnaires organized using Google Forms were sent to pediatric workshop members between March and June 2019. The questionnaire (Figure 1) consisted of questions that measured the participants' basic demographic data, the use of the workshop, and the overall impact of the workshop on their professional behavior. While the institutions and positions of the participants were recorded, no other personal data (such as address and telephone) were collected.

The survey is available at the end of the article (Appendix 1).

## 3. Results

Since the establishment of the Pediatric Atelier Telegram group, 997 pediatric specialists, subspecialists, and specialist-related pediatrics have become involved in this network. A total of 417 (42%) of the members answered the questionnaire. In all, 225 (54%) of these respondents were women, 192 were men, and 241 (58%) were 45 years or under. Respondents included 300 (72%) pediatricians, 21 (5%) pediatric subspecialty fellows, and 75 (18%) pediatric subspecialists (Table 1). Of the 417 respondents, 217 (52%) were working in Istanbul, and 200 (48%) were working in other cities of Turkey.

Among the respondents, 233 (58%) were working in private hospitals or doctors' offices (Table 1). Among the participants, 197% checked in on the Telegram group more than once a day, 117 at least once a day, 51 once a week, and 12 once a month. After the establishment of the Pediatric Atelier Telegram group, 520 cases were consulted in 241 days of the study period (more than 2 consultations per day). There was an upward trend from 9 case consultations in November 2018 to 166 cases in April 2019. Most consultations (*n* = 309, %59) were made from pediatricians working in the Istanbul metropolitan area, 203 (40%) consultations were from other cities of Turkey, and 8 (1%) consultations were from abroad. Most of the consultations (382 (73%)) came from pediatricians working in private hospitals or doctors' offices, 89 (17%) consultations come from pediatricians working in state hospitals, and 49 (9%) consultations come from pediatricians working in universities or teaching hospitals. The most frequently consulted departments were pediatric infectious diseases (166; 32%), pediatric hematology and oncology (56; 11%), neonatology (43; 8%), pediatric endocrinology (40; 8%), pediatric intensive care (36; 7%), and pediatric metabolic diseases (23; 4%) (Table 2). Among the 520 consulted cases, 466 (92%) cases were answered by at least one pediatrics subspecialist, and 43 (8%) were answered by specialists in areas other than pediatrics. A total of 235 (45%) consultations were made during working hours, and 285 (55%) were made during off time. The mean response time to consultation was not different between working hours (20.7 ± 4.7 min) and off time (24.2 ± 8.7 min) (*p* = 0.52). On the other hand, the solution time for consultations was shorter during working hours (38.7 ± 21.3 min) than off time (58.8 ± 13.2 min) (*p* = 0.015). Of the 520 consulted cases, 44 (8%) were related to life-threatening events, 25 were hospitalized in intensive care units, and 6 required surgical operations. Among the 44 patients with acute life-threatening events (ALTEs), 2 died in the intensive care unit. A total of 29 (66%) ALTE cases occurred in the Istanbul metropolitan area, and 15 cases (34%) occurred in other cities. Among 44 ALTE cases, 33 (75%) were treated by pediatricians from a private practice, 4 (9%) were treated in state hospitals, and 7 (16%) were treated in universities or education and teaching hospitals.

When the participants were asked to evaluate whether the Pediatric Atelier group was useful or not, on a scale of 1-10 points, 228 (57%) of them gave 10 points, 74 (18) gave 9 points, 73 (18%) gave 8 points, and 24 (6%) gave 7 seven points. Among all respondents, 82% stated that the case counseling part of the Pediatric Atelier group was the most useful part, and 15% considered the weekly case discussion to be the most important part. Regarding the answers they received for their consultation requests, 281 of the 402 participants (68%) had not sought a consultation, 128 of responders (30%) stated that the problem they asked for a consultation on had been solved, and 8 (2%) stated that the problem had not been solved. Additionally, 50% of participants noted that they regularly followed weekly case reports. The respondents were asked to rate the case consultation interaction on a scale from 1-10 points, 89% of them gave ≥8 points. Additionally, 50% of respondents answered that they regularly follow the live broadcasts. Moreover, 21% of the surveyed members expressed their personal opinions and expressed their wishes for further development of this professional network.

When asked how the members benefit from the Pediatric Atelier group, 320 of 417 (77%) members answered that “I can reach current scientific developments”, 313 (75%) members answered that “It's an extensive and useful professional network”, 290 (69%) members answered that “It facilitates communication between reach academic people and subspecialists”, 288 (69%) members answered that “Case reports provide scientific benefits”, 150 (36%) members answered that “I think it's a good point of reference”, 114 (27%) members answered that “I benefit from live broadcasts”, 94 (22%) members answered that “I use the group while working in an emergency ward”, 46 (11%) members answered that “I use the group for intensive care referrals”, and 43 (10%) members answered that “The Atelier enables me to communicate with my friends” (Figure 1). Among the respondents, all of them recommended the Pediatric Atelier group to their work colleagues.

## 4. Discussion

To the best of our knowledge, this is the largest online pediatric consultation and learning platform accessed by using smartphones. In this medium, we prefer to use Telegram for communication. After the Pediatric Atelier group was founded, real problems about real cases could be discussed, generating specific comments and guidance from field experts and subspecialists with the academic discipline; reference sources for further reading are recommended, and online broadcasts via the YouTube channel (https://www.youtube.com/channel/UCbqCk7V7eNnQ_7v_qmPzFdw) are also posted monthly. Cases requiring intensive care and surgery were evaluated by Pediatric Atelier members specializing in pediatric intensive care, pediatric metabolism, neonatal intensive care, and pediatric surgery. All members worked in solidarity to provide intensive care beds to the patients of the members working in the provinces. The members are composed mainly of pediatricians working in private practices or hospitals where consultation with pediatric subspecialties is difficult. Additionally, the second main group of participants is composed of pediatricians working in rural parts of the country. For this reason, the most preferred area of the Pediatric Atelier group is the case consultation portion. This supports the finding that even in developed countries such as the USA, primary care pediatricians have stated that the most important perceived barriers to accessing pediatric subspecialty and pediatric surgery are long waiting times for appointments, subspecialists not participating in certain health care plans, subspecialists not accepting uninsured patients, the lack of appropriate pediatric subspecialists, and the length of travel time to subspecialists' offices; moreover, these barriers were found to be more prevalent in rural areas than in nonrural areas [6]. For this reason, especially in obtaining pediatric subspecialty consultations more rapidly, online smartphone-based platforms are important. However, these improvements also come with some legal problems. To overcome these problems, we put out legal warning information to all our platform members in the Telegram channel noting that the Pediatric Atelier group was built to share the latest data and professional experience between pediatricians. The comments and suggestions for consulted cases are just the opinions of the doctors expressing them, and such doctors are not legally responsible for the patient; therefore, the decision to follow the suggestion or not still belongs to the pediatrician asking the question. Content on the Internet can overcome physical or temporary barriers, provide retroactively searchable content, and encourage interaction based on printed training tools [3]. Social media applications are online environments where users primarily contribute, receive, and discover content created by other users. Unlike formal written communication technologies, social media content is often created by users, and information is allowed to flow effectively on social media [7]. However, the use of social media by health professionals has raised concerns about patient privacy [8]. In our platform, before sharing the laboratory and the results of patients, verbal consent is obtained from all patients or caregivers.

There are few examples of online or social media usage among health care workers. A survey among oncologists revealed that although the use of social media was intended to be used for educational purposes, more than half of the participants encountered obstacles in accessing the online content, while more than 75% of the members from a young retina forum who responded reported that they interacted with their colleagues at least once a day using the relatively easy Telegram application [4, 5]. In our study, 80% of the surveyed members entered the network at least once a day, and 50% did so more than once a day, probably due to the relatively easy-to-use nature of the Telegram application. As far as we know, there is one known lifelong learning group of urologists using the Telegram application. This urologist group was established in 2014 and is composed of 408 members. This group works through case-based learning discussions and the sharing of clinical experiences, lectures, and evidence-based journal articles [9]. This urologist group and our Pediatric Atelier group show the usefulness of Telegram groups as an exceptionally easy tool that doctors can use to connect to invaluable resources to which access might otherwise be limited by geographic distance or time barriers. This smartphone-based learning platform may be useful in improving clinical knowledge and narrowing the gap between high-quality evidence and clinical practice in developing countries.

On the other hand, there are not enough studies to estimate what kind of information physicians want to obtain through social media platforms [4]. The Pediatrics Atelier group is invitation based; the closed group consists mainly of private practice pediatricians. Most case consultation requests lead to a large group discussion, a review of the current knowledge and management suggestions from experts and subspecialists. Currently, the most common way to obtain this information is through a pediatric workshop, an environment where physicians share their needs in a way that has a continuous, unobstructed, and interactive flow. Our report is the first examination of an interactive pediatric social media group with nearly 1200 active members in Turkey. The Pediatric Atelier group has significant clinical relevance in providing opportunities to improve patient management as well as being an interactive medium of continuous pediatric education. Although it is commonly accepted that young physicians are more interested in engaging in training and cooperation through social media, the Pediatrics Atelier group has active participants of all ages. In the group, there is no significant difference between the number of male and female participants, and it is clear that mobile education through social media and technology has changed the way young physicians learn and practice medicine. The Pediatric Atelier group demonstrates that it is very useful to develop and implement a method for building an online medical community among the relevant pediatric doctors, overcoming the boundaries of knowledge and cooperation through the use of the Telegram application. Despite these benefits, medical education via social media has certain limitations that must be acknowledged. First, the effect of this education program on skills is unclear. Second, there are still some concerns regarding medical ethics and patient privacy and confidentiality in such programs. The limitations of this survey include being subject to potential recall bias or selection bias by having possible preconceived notions regarding SM. Furthermore, because all of the authors were active members of the Pediatric Atelier, our analysis was subject to bias.

In conclusion, to our knowledge, this is the first report to examine how pediatricians professionally experience and perceive using an SM platform. It is obvious that SM and smartphone education through technology are transforming the way in which pediatricians learn and practice medicine. The Pediatric Atelier group shows that developing and implementing a method to build an online medical platform among pediatricians through an SM application such as Telegram is quite beneficial for those involved. In a short period of time without any financial support and advertisement, the Pediatric Atelier group members increased to 1200 through invitations from users. We think that the development of technology and artificial intelligence may lead to the increased usage of online platforms or systems in clinical medical practice.

## Figures and Tables

**Table 1:** Respondent demographics and survey results.

Characteristics	Data *n* (%)
Survey response rate	417/997 (42%)
Gender	
Male	192 (46%)
Female	225 (54%)
Age (years)	
28-35	90 (22%)
26-44	151 (36%)
45-60	125 (30%)
>60	22 (5%)
Speciality area	
Pediatrician	300
Pediatrics subspeciality fellow	21
Pediatric subspecialists	75
Neonatology	18
Pediatric hematology and oncology	9
Pediatric emergency	7
Pediatric intensive care	5
Pediatric allergy and immunology	5
Pediatric endocrinology	4
Pediatric infectious diseases	4
Pediatric cardiology	4
Pediatric gastroenterology	4
Social pediatrics	4
Pediatric nephrology	3
Pediatric metabolism	3
Pediatric neurology	3
Pediatric chest diseases	1
Pediatric rheumatology	1
Others	
Pediatric urology	2
Pediatric surgery	2
Pediatric psychiatry	2
Dermatology	1
Institution	
Private hospitals	190 (49%)
University and teaching hospitals	82 (21%)
State hospitals	78 (21%)
Doctor office	33 (9%)

**Table 2:** Consultations with regard to speciality area.

Speciality	*n*	%
Pediatric infectious diseases	166	32
Pediatric hematology and oncology	56	11
Neonatology	43	8
Pediatric endocrinology	40	8
Pediatric intensive care	36	7
Metabolic diseases	23	4
Pediatric dermatology	17	3
Social pediatrics	12	2
Pediatric nephrology	11	2
Pediatric neurology	11	2
Pediatric cardiology	10	2
Pediatric radiology	10	2
Pediatric allergy	9	2
Child psychiatry	8	2
Pediatric emergency	6	1
Pediatric gastroenterology	6	1
Medicolegal issues	6	1
Pediatric surgery	6	1
Pediatric urology	5	1
Pediatric genetics	4	1
Pediatric orthopedic surgery	4	1
General pediatrics	31	6
	489	%100

## Data Availability

The authors state sharing data available on request through a data access committee, institutional review board, or the authors themselves.

## Appendix

Pediatric network questionnaire;

Pediatric Workshop Member Physicians Recognition Form

Questions:

**Your Gender?** Female Male

**Mark the age group that suits you;** 23-2728-3536-4446-6061 and above

**What is your academic title?** Prof. Dr. Assoc. Prof. Dr. Faculty Member Fellow Specialist

**What are your total years of expertise?** 1-56-1011-1515 and above

**What is your mandatory service obligation status?**

I work as a compulsory service I have a post-specialty obligation

My compulsory service obligation is over I am in a lucky group with no obligatory service

**Did you / did you receive subspecialty training?**

I am general pediatrician. I have completed my subpecialization training

I'm fellow at. ….. subspecialty. I did not complete the subspecialty training

**What is your area of expertise?**

Pediatric Emergency/ Pediatric Endocrinology/ Pediatric Infectious Diseases/ Pediatric Gastroenterology, Hepatology and Nutrition/ Pediatric Genetic Diseases/Pediatric Pulmonology/ Pediatric Hematology and Oncology/ Pediatric Immunology and Allergy /Pediatric Cardiology/ Pediatric Metabolic Diseases/ Pediatric Nephrology/ Pediatric Neurology/ Pediatric Radiology/ Pediatric Rheumatology/ Pediatric Intensive Care/ Developmental Pediatrics/Social Pediatrics/ Neonatal Intensive Care/ General Pediatrics/ Pediatric Surgery/ Child Psychiatry/ Pediatric Dermatology/ Pediatric Otolaryngology/ Pediatric Urology/ Pediatric Sports Medicine

**Where do you work?**Province - (Town),

**Institution** University and Education Research Hospital/Public Hospital/ Private Hospital or Medical Center

**How often do you look at the pediatric workshop telegram group?**

Once a month or less Once a week Once a day 1-5 times a day I look at it often during the day

Just looking at case reports At live broadcasts Looking for the answer when I'm going to consult the case

**Do you find the pediatrics workshop useful?** Yes No

**If you had to score about the benefit of the Pediatrics Workshop, how many would you give? (1st worst, 10 best)**

1. Useless 2. 3. 4. 5. 6. 7. 8. 9. 10. very helpful

**What do you think is the most useful part of the pediatrics workshop?**

Case counseling / asking questions Weekly case report Monthly live broadcast

**If you gave 1-10 points to the case consultation, how many would you give? (1st worst, 10 best)**

1. Waste of time 2. 3. 4. 5. 6. 7. 8. 9. 10 Very helpful

**How often do you follow the weekly case report?**

Every week 2-3 times a month 1 or less per month I never watched

**How often do you watch monthly live streaming?**

I've watched only one or more than 1 time I did not watch

**Which way do you find useful? Please tick the appropriate options.**

An extensive and useful professional network

Makes it easy for me to reach academic people and subspecialists

I can learn current scientific developments Facilitates intensive care and surgical referrals

I can use it when my head is stuck in seizures and polyclinics

I benefit from case reports I benefit from live broadcasts

Allows me to communicate with my friends Good reference point

**Has the pediatrics workshop changed your clinical approach? Can you share if it happened?**

Yes No

**Have you consulted a case or idea with the Pediatrics Workshop? Have you consulted the outcome of the case, was it positive for you and the situation?**

I do not consult any case. I consulted; my problem solved I consulted, my problem is not solved

**Would you recommend the Pediatrics Workshop to your friend?** Yes No

**Thank you for participating in our survey. We would be very happy to write your suggestions for the development of the Pediatrics workshop.**

## Abbreviations

SM: Social media.

## Additional Points

*Table of Contents Summary*. This article describes a social networking experience that pediatricians use for case counseling and postgraduate education. *What's Known on This Subject*. Social media applications are used in various medical specialities for postgraduate education, patient, and disease management counseling. *What This Study Adds*. The consultation and referral of patients and disease management through the application of telegram with a large community of specialists and subspecialists in pediatrics seems promising. Sharing information with such application groups will enable our colleagues working far from the center to work more comfortably.
